# Evaluation of physical health assessments for new admissions to the Oleaster during the first wave of COVID-19

**DOI:** 10.1192/bjo.2021.269

**Published:** 2021-06-18

**Authors:** Erin Lawson-Smith, Danielle Sutherland, Eleanor Brookes, Alex Zhang, Joji George

**Affiliations:** 1 University of Birmingham, Medical School; 2 Birmingham and Solihull Mental Health Foundation Trust

## Abstract

**Aims:**

Physical health of psychiatric inpatients is worse than the general population. Physical health monitoring of these patients can have positive effects on outcomes. Birmingham and Solihull Mental Health Foundation Trust (BSMHFT) states that a physical health assessment (PHA) should be completed within 72 hours of admission. This comprises a physical health form (PHF) and minimum data set (MDS): BP, BMI, TB and BBV status, alcohol and drug screen, smoking status, Hba1c and lipids. In a 2017 audit, compliance was shown to need improvement, with 28.3% of admissions not having a PHF documented.

**Objectives:**

To assess whether PHAs for new admissions to the Oleaster, Birmingham during the first wave of COVID-19 were completed in line with trust policy

To compare findings with a previous audit

To make recommendations to improve inpatient physical health and compliance with trust policy

**Method:**

A retrospective audit was conducted, with PHA details accessed via the electronic medical records system RiO. Admissions from 16/03/2020-30/06/2020 were accessed and 158 admissions (155 patients) were included. 21 admissions were excluded as they were internal transfers; only data from the initial admission were included. Data were collected by 2 medical students and a psychiatry trainee using a data collection tool. Data were recorded and analysed on Excel.

**Result:**

Of 158 admissions, 81 had PHFs (51.3%). 59 were completed within 72 hours of admission (34.3%); 39 were completed fully (24.7%). Of incomplete PHFs, 2 explicitly stated incompletion due to COVID-19. 22 PHFs were created but not completed within 72 hours. 15 gave a deferral reason e.g., refusal to consent or agitation. For 77 admissions (47.3%), no assessment was documented, with no reason given.

2 admissions (1.3%) recorded the full MDS within 72 hours of admission.

2 admissions (1.3%) had fully complete PHAs (PHF and MDS) within 72 hours of admission, fulfilling trust policy.

**Conclusion:**

51.3% of admissions had a PHF, with 34.3% documented within 72 hours of admission. However, only 1.3% of admissions fulfilled trust policy of both a completed PHF and MDS within 72 hours of admission. There were more admissions without a PHF than in the previous 2017 audit; 47.33% compared to 28.3% previously. Given trust targets that a PHA should be fully completed for 100% of admissions, it was found that the Oleaster did not meet these guidelines during this period and improvements must be made to maintain integrity of patient care.

